# Validating a novel capability of assessing pathways of animal water gain and loss

**DOI:** 10.1098/rsos.241942

**Published:** 2025-05-21

**Authors:** Zachary T. Steele, Karen Caceres, Zachary A. David, Lisa M. Shollenberger, Alexander R. Gerson, Seth D. Newsome, John P. Whiteman

**Affiliations:** ^1^ Biological Sciences, Old Dominion University, Norfolk, VA, USA; ^2^ Wildlife Ecology and Conservation, University of Florida, Gainesville, FL, USA; ^3^ Biology, University of Massachusetts Amherst, Amherst, MA, USA; ^4^ Biology Department, The University of New Mexico, Albuquerque, NM, USA

**Keywords:** triple oxygen isotopes, doubly labelled water, metabolic water, stable isotopes, ^17^O-excess, animal water balance

## Abstract

Understanding variations in the routes by which wild animals gain and lose water is challenging, and common methods require longitudinal sampling, which can be prohibitive. However, a new approach uses *Δ*′^17^O_BW_ (*Δ*′^17^O of animal body water), calculated from measurements of *δ*′^17^O and *δ*′^18^O in a single sample, as a natural tracer of water flux. *Δ*′^17^O_BW_ is promising, but its relationship to organismal variables such as metabolic rate and water intake have not been validated. Here, we continuously measured oxygen influxes and effluxes of captive deer mice (*Peromyscus maniculatus*), and manipulated their water intake and metabolic rate. We used these oxygen flux data to predict *Δ*′^17^O_BW_ for the mice and compared these model predictions with *Δ*′^17^O_BW_ measured in blood plasma samples. As expected, *Δ*′^17^O_BW_ positively correlated with drinking water intake and negatively correlated with metabolic rate. All predicted *Δ*′^17^O_BW_ (based on measured oxygen fluxes) values differed from measured *Δ*′^17^O_BW_ values by <30 per meg (mean absolute difference: 11 ± 9 per meg), suggesting high accuracy for this modelling approach because studies currently report a range of 300 per meg for *Δ*′^17^O_BW_ among mammals, birds and fish.

## 1. Introduction

Water and energy are essential to all animals, and these resources are linked via the biochemical mechanisms that sustain life. As heterotrophs, animals obtain their energy by catabolizing the organic matter in their diet [1]. This catabolism—and the anabolism to build and repair tissue—requires an aqueous setting, while also producing and consuming water [2]. The ubiquity and importance of water in animal biology means that negative water balance, in which loss exceeds input, can hinder an animal’s performance and threaten its survival.

While the potential pathways by which animals obtain water and energy are similar across species, the contribution to the total body water pool from each source varies considerably based on taxa, life stage and habitat [3–5]. Many animals primarily rely on intake of exogenous, pre-formed water via drinking, their food or unique pathways such as absorption through the integument. Pre-formed water is also commonly referred to as meteoric water because this water is ultimately sourced from precipitation [1,3].

Animals also perform endogenous, *de novo* synthesis of water. Metabolic water commonly refers to water produced by two (often linked) biochemical processes: the actions of cytochrome c oxidase and dehydration–condensation reactions [6,7]. Cytochrome c oxidase is the final complex of the mitochondrial electron transport chain, and it combines atmospheric oxygen with hydrogen via electron transfer to yield new H_2_O molecules (hereafter ‘oxidase water’ [1,6]). Dehydration–condensation reactions generate water as a byproduct of combining reactants, such as enolase catalysing the conversion of 2-phosphoglycerate to phosphoenolpyruvate during glycolysis [2]. Catabolizing food for energy yields this ‘condensation water’, in which an oxygen atom that was bound in the catabolized substrate is transferred to an H_2_O molecule [2]. Dehydration–condensation reactions occur in many biochemical pathways other than food catabolism, like the conversion of adenosine diphosphate to adenosine triphosphate (ATP) via ATP Synthase; however, such reactions do not incorporate new, exogenous oxygen atoms (i.e. from ingested food) into water, thus they essentially recycle oxygen already present in the body [2,6]. Here, we use ‘condensation water’ only to refer to water synthesized from oxygen derived from food.

Regardless of the process by which water is obtained, animals balance these inputs with outputs such as the loss of water vapour during exhalation of breath, or loss of liquid water in excretion of nitrogenous waste via urine [3]. Water loss via urine and faeces are typically substantial outputs [3], and the ability for an animal to minimize water loss via these outputs may be a critical tool for predicting expected impacts of global climate change on different species [8]. This ability to manipulate outputs and thus conserve water, varies dramatically among species [8]. For example, some desert species have specialized kidneys that enable excretion of highly concentrated urine, minimizing water loss [9].

Water is critically important for wild animals, and it can shape their behaviour and population trends [10]. For example, wildebeest (*Connochaetes taurinus*) of the Serengeti migrate 500 km or more to meet their water needs, and the increasing frequency of droughts has resulted in significant population declines [11,12]. Despite the importance of water in ecology and conservation—and the importance of the closely related processes of energy flux—few methods are currently available for assessing the water and energy balance of free-ranging animals [7,13]. Existing methods rely on stable isotope analysis (SIA) of oxygen and hydrogen [5,7]. SIA data is reported in delta (*δ*) notation based on relative differences of ratios of heavy-to-light isotopes between the sample and internationally accepted standards, which for oxygen and hydrogen are Vienna Mean Standard Ocean Water (VSMOW) and Standard Antarctic Light Precipitation Water (SLAP) [14].

The most common method using SIA to assess water and energy metabolism in free-ranging animals is the doubly labelled water (DLW) approach that involves injecting tracers enriched in ‘heavy’ ^2^H and ^18^O [5,15]. The decrease of these enriched tracers in animal body water at subsequent sampling events reflects the fluxes of CO_2_ and H_2_O, providing a means to estimate water turnover and metabolic rate [5,15]. Water inputs and outputs can also be estimated with measurements of the natural abundance of *δ*^18^O in body water (*δ*^18^O_BW_) without the use of enriched tracers [3,16]. The value of *δ*^18^O_BW_ reflects the integration of the oxygen influxes (e.g. drinking water) and effluxes (e.g. exhaled CO_2_) to and from the body water pool, and *δ*^18^O_BW_ models have been developed to interpret the fractional contributions from these different fluxes [3,16–18]. However, the *δ*^18^O of potential water sources can be highly variable across space and time because of the ubiquity of mass-dependent fractionation processes, often making it difficult or impossible to constrain model inputs and draw meaningful inferences, especially for wide-ranging animals.

Promising recent studies may provide a better understanding of animal water balance through measurement of both ^17^O and ^18^O. This approach is based on the relationship between *δ*^17^O and *δ*^18^O, which has a near constant slope of 0.528 [19,20] because mass-dependent fractionation affects both isotopes, meaning that a material with a high *δ*^18^O will also have a high *δ*^17^O value [14]. Two processes can cause *δ*^17^O to deviate from this relationship. First, some mass-dependent fractionation processes (e.g. diffusion of water vapour through air) result in a slope different than 0.528 for the relationship between *δ*^17^O and *δ*^18^O. Second, mass-independent fractionation can also occur, causing *δ*^17^O to vary in a manner unrelated to the slope. Both these processes result in deviation in the relationship between *δ*^17^O and *δ*^18^O that is quantified as *Δ*′^17^O [20,21]:


(1.1) $$\Delta^{\prime 17}O=\delta^{\prime 17}O-0.528{\;}^{*}\;\delta^{\prime 18}O$$

In equation (1.1), *δ*^17^O and *δ*^18^O are linearized with the relationship *ln* * (*δ*^*x*^O + 1), where *x* is 17 or 18. Since many mass-dependent fractionation processes follow a slope of 0.528, and because mass-independent fractionation is rare, *Δ*′^17^O typically exhibits far less variation than *δ*^18^O. Thus, *Δ*′^17^O can be a powerful tracer because pre-formed water has a relatively consistent value of 0−40 per meg (parts per million; 0−0.040‰; [21,22]). By contrast, oxidase water is formed using inhaled atmospheric oxygen atoms, which have a *Δ*′^17^O of −441 per meg [23] due to the fractionating processes associated with ozone formation in the stratosphere [24]. Furthermore, the synthesis rate of oxidase water is a direct reflection of metabolic rate [25], thus *Δ*′^17^O_BW_ should be influenced by metabolic rate [7].

Measurements of *Δ*′^17^O_BW_ may be useful for understanding the ecophysiology of free-ranging animals [7,26–29], and for informing paleoclimate reconstruction based on fossilized animal remains [22,30]. Thus far, *Δ*′^17^O_BW_ has been inferred from measurements of eggshells, bones and teeth [22,26,27,30], as well as directly measured in body water distilled from blood [7,28,29,31]. To interpret *Δ*′^17^O_BW_ measurements, studies have used mass balance models that allocate fractional water input to a suite of potential sources such as drinking and food water versus oxidase water [7]. This approach is limited because the predictors are not clearly linked in a biological context, nor are the avenues of water efflux accounted for, which could influence *Δ*′^17^O_BW_. Recently, flux-based models have been described, with predictors that reflect all of the potential oxygen inputs and outputs [22,27]. Inclusion of these additional parameters adds biological realism but also creates challenges because many of these parameters require estimating unknown values, including fractionation factors that may affect *δ*^18^O (^18/16^*α*) or *Δ*′^17^O (*θ*). Predictions from flux-based models of *Δ*′^17^O_BW_ have yet to be compared with *Δ*′^17^O_BW_ values measured in captive animals for which oxygen fluxes can be directly measured.

Here we predicted *Δ*′^17^O_BW_ values for captive deer mice (*Peromyscus maniculatus*) by measuring multiple oxygen inputs and outputs and applying these data to a modified flux-based model [22]. We then compared model predictions with measurements of *Δ*′^17^O_BW_ in distilled blood samples. We tested assumptions about how physiological processes influence *Δ*′^17^O_BW_ by manipulating and measuring the inputs of oxidase water (via alterations of metabolic rate) and pre-formed water (via alterations of drinking water intake). We predicted that: (i) increased drinking water intake would cause a higher contribution from exogenous, pre-formed water, elevating *Δ*′^17^O_BW_; and (ii) increased metabolic rate would cause a higher contribution from oxidase water, lowering *Δ*′^17^O_BW_.

## 2. Material and methods

### 2.1. Study site and animals

Eight female deer mice were purchased from the Peromyscus Genetic Stock Center at the University of South Carolina (Columbia, SC, USA). Upon arrival to Old Dominion University (ODU; Norfolk, VA, USA), mice underwent health inspections then were transferred to cages for single housing (21 × 37 × 14 cm; Mouse Respirometry Cage; Sable Systems International (SSI), NV, USA), containing bedding and nesting material and three ‘hoppers’: (i) a feeder (Food Intake Monitor; SSI), (ii) a drinker (Water Intake Monitor; SSI) and (iii) a covered resting platform (Body Mass Monitor; SSI). These eight cages, and one empty baseline cage (a carboy with a similar volume as the cages), were inside a temperature control cabinet (Model 7000-25-1; Caron Products & Services, Inc., OH, USA) set to 25°C unless otherwise noted.

### 2.2. Metabolic phenotyping system

Each cage was connected to a Promethion metabolic phenotyping system (SSI). A metal tube with small holes ran along the bottom perimeter of each cage and was connected to a pump and respirometry equipment via tubing fitted with a high-efficiency particulate air filter. Air was continuously drawn out of each cage at 2000 ml min^−1^. Air from a single cage at a time was directed through CO_2_, O_2_ and H_2_O sensors. Each individual cage measurement lasted 30 s and four cages were measured sequentially, followed by a 30 s baseline cage measurement. Therefore, a full cycle of eight cages lasted 5 min, with each cage measured once every 5 min. Additionally, each hopper was suspended from a sensor (MM2 Load Cell) that recorded the mass every 2 s with precision of 0.002 g. When an animal consumed food or water, the change in mass reflected the food or water consumption, and similarly, whenever the animal entered the resting platform, the monitor detected the mass of the animal.

A required daily reboot and calibration of the software (which was subsequently resolved with updates), along with sampling efforts and equipment maintenance, occasionally resulted in data gaps of 35−335 min. We filled in this gap with the average O_2_ intake and CO_2_ output from the three previous hours when the gap was 35−60 min (this occurred on 16 days), and from the previous 24 h when the gap was >60 min to account for likely elevated O_2_ intake and CO_2_ output caused by the increased presence of researchers (this occurred on 8 days). Automated mass-monitoring of drinkers has been reported to overestimate water intake by 1−13% because of drippy nozzles, spills during drinking or spills from the animal bumping the nozzle [32,33]. To account for this bias, we reduced all water intake values in our study by 10% because mice often built nests directly under the water hopper, which may have resulted in nozzle leakage. Daily recordings of water or food intake with unrealistic values indicative of substantial water spillage (e.g. 25 ml intake for an 18 g mouse) or food caching (e.g. 30 g intake for an 18 g mouse) were replaced with a calculated average (electronic supplementary material, table S1).

### 2.3. Study design

We tested how *Δ*′^17^O_BW_ values responded to increases in metabolic rate and in water intake. We increased the metabolic rate by lowering the housing temperature from 25°C to 15−18°C, below the lower critical temperature of the mice (25–30°C [34,35]), which we expected would induce thermogenesis. We increased the salt content of their diet from 0.49% NaCl (Teklad Diet no. TD.96208; Inotiv, West Lafayette, Indiana, USA) to 4% NaCl (Teklad Diet no. TD.92034; Inotiv; nearly identical nutrient composition to TD.92608; see electronic supplementary material, table S2), which we expected would increase water intake for osmoregulation. Mice were separated into Group A and Group B, and all mice experienced each combination of control and cold temperatures, and 0.49% NaCl and 4% NaCl diet.

**Figure 1.**
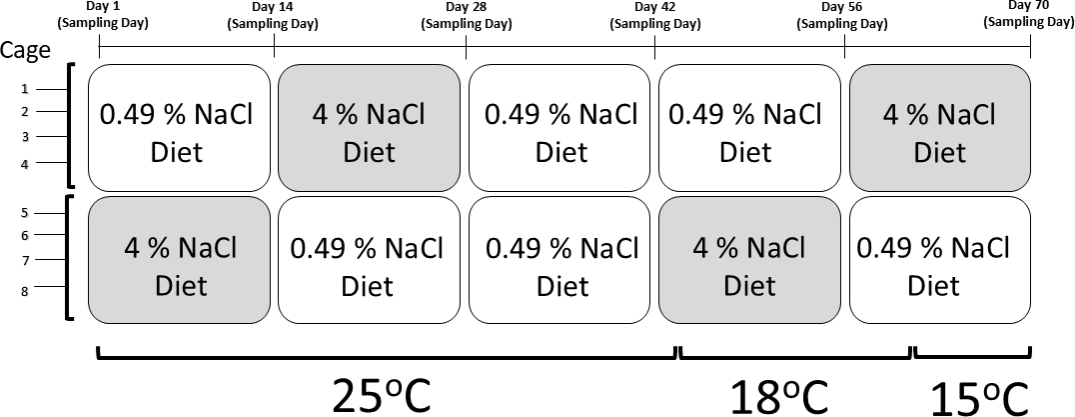
Experimental design for eight captive deer mice (one per cage) experiencing manipulations in housing temperature and dietary salt content. Days 28−42 were a ‘reset’ period to allow animals to recover from consuming the 4% NaCl (high salt) diet, prior to reducing housing temperature for the remainder of the experiment. Blood sampling occurred every 14 days. Housing temperature reached 18°C on day 44 and 15°C on day 64.

Before the experiment, mice were acclimated for 41−156 days to eliminate acclimation effects in critical variables such as their oxygen utilization fraction (OUF) [36–38], which represents the fraction of O_2_ absorbed during a typical breath compared with the O_2_ content in ambient air (also referred to as oxygen extraction efficiency). During the experiment, each manipulation lasted 14 days, including a 14 day ‘reset’ period on days 28−42 (figure 1). During days 1−42, the temperature cabinet was set to 25°C. Beginning on day 42, the temperature was reduced at a max rate of 3.5°C day^−1^, with the goal of reaching 15°C. To keep relative humidity <70 %, desiccants were added to the temperature cabinet and temperature was held at 18°C during days 44−61, before reaching 15°C on day 64.

### 2.4. Sample collection and stable isotope analysis

On day 1 and on the final day of each manipulation (except day 70 because animals were euthanized), blood samples (≤200 μl) were collected via facial vein bleeds using a small lancet (Goldenrod 5 mm Animal Lancet) and collection tube (Sarstedt 200 µl Microvette [39–41]). On the final day of the experiment (day 70), blood samples were collected via a perimortem cardiac puncture [42,43]. Immediately after sample collection, the plasma was separated via centrifugation, except for samples below 100 μl, which were left as whole blood for distillation.

Plasma (or whole blood) was cryogenically distilled to obtain a body water sample, following the methods in Steele *et al*. [31]. In brief, we transferred 25−45 μl of sample into the large end of a 9-inch glass Pasteur pipette, then flame-sealed this end with a mini blowtorch. We froze the sample with liquid nitrogen, created a vacuum inside the pipette and flame-sealed the narrow end, then gently warmed one end of the sealed tube, driving water to evaporate from the sample and condense at the other end of the tube. Careful monitoring of the quality of flame-seals on the glass pipette minimizes unintentional fractionation during distillation, typically resulting in variation among sample replicates of ≤0.3‰ for *δ*^18^O_BW_ and ≤15 per meg for *Δ*′^17^O_BW_ [31]. Once distilled, samples were transferred into a 400 μl insert within a 2 ml autosampler vial. Each distilled replicate was injected and measured 5−11 times on a water isotope analyser (see below), depending on volume.

Oxygen and hydrogen stable isotopes were measured on a Picarro L2140-*i* (Picarro, Inc., CA, USA) in ‘analysis runs’ composed of samples, two internationally accepted water standards (USGS50 and USGS47 [44,45]) that were previously validated using VSMOW and SLAP, and ≥5 control waters that had also previously been measured against VSMOW-SLAP. Standards bracketed the potential *δ*^18^O values of samples [31,46]. Control waters were used to track accuracy and precision among runs and to bridge gaps in *δ*^18^O values between samples to reduce memory effects of the instrument [31,46]. The USGS (United States Geological Survey) standards were used to calculate stretching and offset values to determine correction factors for *δ*^17^O, *δ*^18^O and *δ*^2^H, which in turn were used to correct raw measurement values provided by the Picarro L2140-*i* [14,46]. We applied stretching and offset values using slope-intercept form (corrected value = (stretching value * raw value) + offset value) to all measurements, then calculated *Δ*′^17^O using corrected values. We then determined a mean and standard deviation across the measurements for each water analysed (e.g. standards, control waters, samples), removed outliers, and re-calculated the mean, using an automated post-processing script [31] in R version 4.3.1 [47]. After data processing, we reviewed the finalized dataset for each analysis run and excluded datasets if >2 of the control waters’ measured mean value differed from their known value by these thresholds: *δ*^17^O≥0.15 ‰, *δ*^18^O≥0.3 ‰, *Δ*′^17^O≥15 per meg.

Sample volume was sufficient for 1−5 distillations per sample (electronic supplementary material, table S3). When multiple distilled replicates were measured for an individual sample, a mean was calculated across these distillations, leading to a grand mean for that sample [31]. We did not statistically weight datapoints because more measurements do not necessarily mean the data were of greater quality because the accuracy and precision could vary between analysis runs.

### 2.5. Isotope modelling

The final *Δ*′^17^O_BW_ value for each individual sample was compared with the *Δ*′^17^O_BW_ value predicted by a modified version of the flux-based model described by Hu *et al*. [22], which was derived from the *δ*^18^O_BW_ model by Kohn [3]. The Hu *et al*. [22] model used parameters such as body mass, dietary components and metabolic scaling to estimate inputs and outputs of moles of oxygen. We replaced estimates with measurements for several variables, generated estimates for other variables, and additionally constrained other variables as described below (tables 1 and 2).

**Table 1.** List of model parameters and values for ‘base model’ used for sensitivity analysis. Values for the base model were obtained using the 48 h, 25°C, control model parameters in combination with average deer mice values for remaining parameters.

parameter	value
mass (kg)	0.02^a^
faecal H_2_O content (%)	55^b^
oxygen utilization fraction/fraction of O_2_ used (%)	25^c^
*z*-value (‰)	10.5^d^
body temperature (C)	38^e^
Meeh factor (*k*)	1100^d^
skin H_2_O evaporation rate (mg/cm^2^/h/mm Hg)	0.025^f^^,g^
O_2_ inhaled (ml)	5582^a^^k^
CO_2_ exhaled (moles)	0.21417^a^^k^
relative humidity (%)	45^f^
environmental temperature (C)	0.25^f^
pre-formed water *δ*^18^O (‰)	−4^f^
pre-formed water *Δ*′^17^O (per meg)	20^f^
atmospheric O_2_ *δ*^18^O (‰)	24.046^h^
atmospheric O_2_ *Δ*′^17^O (per meg)	−441^h^
food consumed (kg)	0.01297^a^^k^
drinking water intake (ml)	10.44^a^^k^
relative digestibility (%)	85^d^
energy extraction efficiency (%)	100^d^
water nozzle leakage (% of water intake estimated to be leakage)	10^f^
food associated free H_2_O content (%)	9^f^
food carbohydrate content (% by weight)	67.2^f^
food protein content (% by weight)	25.4^f^
food fat content (% by weight)	7.4^f^
food *δ*^18^O (‰)	−16.41^f^^,i^
food *Δ*′^17^O (per meg)	30^f^^,i^
*δ*^18^O of the cellulose of corn ingredients in diet (‰)	26^j^
*δ*^18^O of the cellulose of wheat ingredients in diet (‰)	26.9^j^
*δ*^18^O of the cellulose of soybean ingredients in diet (‰)	25.9^j^

^a^ Average value from deer mice data.

^b^ [48].

^c^ Derived from Rezende *et al*. [36] after acclimation.

^d^ [3].

^e^ [49,50].

^f^ Standard value for mice using the 48 h 25°C, control model parameters.

^g^ Value listed by Schmidt-Nielsen [51] for similar *Peromyscus* species; calculated transcutaneous water loss using this value is comparable with similar studies in other rodents (see [52–54]).

^h^ Measured value listed in Wostbrock *et al*. [23].

^i^ Calculated assuming equilibrium with water vapour; see §2.

^j^ Calculated using assumptions; see §2.

^k^ Not a model parameter; only included to allow model sensitivity testing.

**Table 2.** List of model oxygen fluxes.

inputs	outputs
drinking water^a^	bound oxygen in faecal waste^c^
food water^a^	faecal water loss^b^
oxidase water^a^	transcutaneous water vapour loss^c^
inhaled water vapour^c^	oral water vapour loss^b^
condensation water^c^	nasal water vapour loss^b^
decarboxylation oxygen^c^	exhaled CO_2_^a^
	oxygen bound in excreted urea^b^
	urinary water loss^d^

^a^ Measured using metabolic phenotyping data.

^b^ Estimated based on previous studies.

^c^ Estimated based on metabolic phenotyping data.

^d^ Calculated based on other inputs and outputs.

### 2.6. Model inputs

Our flux-based model included six oxygen inputs (table 2), each of which was defined by moles of oxygen over the time period of measurement (which varied by manipulation), *δ*^18^O and *Δ*′^17^O of the oxygen source, and fractionation that could have occurred during input (^18/16^*α*, ^17/16^*α* or *θ*). The first input was moles of drinking water, which was a measured value adjusted for drinker spillage as described above and converted from mass (H_2_O: 18 g per mol). The measured *δ*^18^O and *Δ*′^17^O of the mice drinking water was −4.0 ‰ (s.d. ± 0.01 ‰) and 20 per meg (±4 per meg) respectively. No fractionation factors were applied to this input.

Second, moles of food water input were calculated from measurements of the mass of food consumed (adjusted for caching, when necessary, as described above):


(2.1) $$\text{Food consumed}(kg){\;}^{*}\;55.56/(1–WC){\;}^{*}\;WC$$

The value WC is the water content by mass of the diet, which was 0.09 (Teklad 2023, personal communication), while the value of 55.56 represents the conversion from kg to moles. We assumed that food water was in isotopic equilibrium with atmospheric water vapour because the food was exposed to ambient air in the rodent cages. We estimated the atmospheric water vapour *δ*^18^O during the time frame of the experiment [55]: May *δ*^18^O: −18.16‰; June *δ*^18^O: −16.41‰; and July *δ*^18^O: −16.35‰. Additionally, we assumed a *Δ*′^17^O of 30 per meg [56–58]. No fractionation factors were applied to this input.

Third, we determined the oxygen input of oxidase water. We used the measured total oxygen consumption (in ml); assumed that each ml contained 5.38 * 10^19^ oxygen atoms (based on the ideal gas law); and assumed that each oxygen atom was incorporated into a molecule of oxidase water [7]:


(2.2) $$\text{Oxygen intake}\;(ml){\;}^{*}\;(5.38{\;}^{*}\;10^{19})/(6.03{\;}^{*}\;10^{23})$$

An ^18/16^*α* value for the synthesis of oxidase water was calculated as:


(2.3) $$\left(1000+(\delta^{18}\;O_{air}–Z)/(1–OUF))/(1000+\delta^{18}\;O_{air}\right)$$

For *δ*^18^O_air_, we used 24.046 ‰ for *δ*^18^O (and elsewhere, when relevant, −441 per meg for *Δ*′^17^O_air_ [23]), for Z we used a ‘*z*-value’ of 10.5‰, and for OUF we used 0.25 (i.e. 25%) [3,36]. The *z*-value represents fractionation of *δ*^18^O that occurs during respiration by comparing the *δ*^18^O of breath samples and the *δ*^18^O of atmospheric oxygen [59,60].

After calculating an ^18/16^*α* value, a *θ* value of 0.5179 (based on respiration of various organisms [61]) was used to calculate the ^17/16^*α* value via the following equation:


(2.4) $$e^{\left(\theta {\;}^{*}\;ln(\delta^{18}\;O{\;}_{\text{fractionation factor}})\right)}$$

Fourth, for intake of atmospheric water vapour, we converted the ml of atmospheric O_2_ consumption to moles, and then calculated the associated amount of air that would be fluxed through the lungs (in l) using the following [3]:


(2.5) $$\text{Oxygen intake (moles)}\;/\;OUF\;/0.21{\;}^{*}\;22.4$$

In this equation, 0.21 is the fraction of oxygen in the atmosphere and 22.4 is the conversion of moles of oxygen to litres. The air fluxed through the lungs was then combined with the relative humidity and saturation concentration of water in air to estimate atmospheric water vapour intake in moles [3]:


(2.6) $$\text{Relative humidity}{\;}^{*}\;10^{\left(0.686+0.027\;*\;(AmbientTemp[K])\right)}/\;760\;/\;22.4{\;}^{*}\;l\;air$$

An ^18/16^*α* value for the intake of atmospheric water vapour was calculated as follows [62,63]:


(2.7) $$e^{\left(11.36{\;}^{*}\;10^{5}/Ambient\;Temp[K{]}^{2}–4.2{\;}^{*}\;10^{2}/Ambient\;Temp[K]–2.07)/1000\right)}$$

After calculating this ^18/16^*α* value, the *θ* value of 0.529 [64] was used to calculate the ^17/16^*α* value using equation (2.4).

The fifth oxygen input was condensation water. We assumed that the primary carbohydrate (and not proteins or lipids; see §4) of glucose provided bound oxygen for synthesis of condensation water, and that two of the six bound oxygen atoms in each glucose appeared in condensation water, with the remaining four appearing in CO_2_. We then calculated the moles of bound oxygen per kg of food using the fractional content of diet by mass that was composed of carbohydrates (67.2% for the 0.49% NaCl diet, and 65.8% for the 4% NaCl diet) and multiplied this by the moles of oxygen per kg of carbohydrates (11.12). The value of 11.12 was determined using the molar mass of glucose (180 g / mol) converted to kg. This provided a value of 5.56, which we then doubled to represent the two (of six) oxygen atoms incorporated per molecule of glucose (thus 11.12). We then factored in the efficiency of assimilation to determine the total moles of bound oxygen using the following [3]:


(2.8) $$\text{food consumed(kg)}{\;}^{*}\;\text{moles of bound oxygen per kg of food}{\;}^{*}\;D{\;}^{*}\;E$$

In this equation, *D* represents the estimated digestibility of this diet for the animal, defined as the ratio of the amount of the food item that is absorbed compared with the amount consumed, while *E* represents the estimated energy extraction efficiency, defined as the ratio of the amount of energy assimilated versus energy potential of absorbed food.

To estimate the isotope values of the bound oxygen in food, we used the midpoint of the estimated range of *δ*^18^O of cellulose for the main ingredients in the 0.49% NaCl diet (TD.96208; corn = 43%; wheat = 37%; and soybean = 20%) and in the 4% NaCl diet (TD.92034; 39%; 39%; 22%). These ranges consisted of the maximum and minimum cellulose *δ*^18^O values based on relative humidity and temperature during harvest in the region where the food was produced (Teklad is based in Wisconsin, USA), using the model of Roden *et al*. [65]: corn in July–September (12–28°C, 71−77% rh), wheat in July (17–28°C, 71% rh) and soybean in October–November (−1–15°C, 73−77% rh). This approach resulted in midpoint estimates of cellulose *δ*^18^O values for corn = 26‰; wheat = 26.9‰; and soybean = 25.9‰. Using these estimated cellulose *δ*^18^O values, we then calculated an ^18/16^*α* value for corn, wheat and soybean using the following equation:


(2.9) $$\left(1000+cellulose\;\delta^{18}\;O[‰])/(1000+\text{pre-formed water}\delta^{18}\;O[‰]\right)$$

After calculating each of these ^18/16^*α* values, a ^17/16^*α* value was calculated for each using a *θ* value of 0.525 [30] and applying each ^18/16^*α* value to equation (2.4).

The sixth and final oxygen input was CO_2_. As described above, when glucose is oxidized for energy, four of the six bound oxygen atoms appear in CO_2_. Prior to loss via exhalation, those CO_2_ molecules exchange oxygen atoms with body water, primarily via the bicarbonate buffer reactions that are catalysed by carbonic anhydrase [66] (see §4). As a result of this exchange, 67% of the bound oxygen in dietary glucose, and all the bound oxygen in lipid and protein, is an input to body water in the form of CO_2_, hereafter referred to as ‘decarboxylation oxygen’. The calculation of this input is:


(2.10) $$f_{Carb}\;{\;}^{*}\;22.24+f_{Lipid}\;{\;}^{*}\;4+f_{Protein}\;{\;}^{*}\;6$$

In this equation, the fractional contribution of each type of dietary macromolecule (carbohydrates, lipids and proteins) by mass is multiplied by the amount of moles of oxygen per each component, and then summed together. Like the calculation of the moles of oxygen related to condensation water, this value is then multiplied by the estimated digestibility and energy extraction efficiency to calculate the moles of decarboxylation oxygen. Since decarboxylation oxygen is sourced from diet like condensation water, we assumed the same isotope values, *θ* value, and ^18/16^*α* and ^17/16^*α* values (see equations (2.4) and (2.9)).

We summed these six oxygen inputs and calculated the fractional contribution of each (e.g. drinking water/total oxygen input). We calculated absolute ratios of ^18^O/^16^O and ^17^O/^16^O for pre-formed water, atmospheric oxygen and bound oxygen in the diet using *δ*^18^O and *Δ*′^17^O for each of these oxygen sources [22]. Then, the fractional contributions were multiplied by their respective ^18/16^*α* and ^17/16^*α* values, and each of these were summed together based on which oxygen source they were derived from (i.e. atmospheric oxygen, bound oxygen or pre-formed water) and multiplied by the absolute ratio of that oxygen source. For example, for atmospheric oxygen ^18^O the calculation of inputs was:


(2.11) $$\begin{array}{r} {\text{Absolute ratio of atmospheric oxygen}}^{18}O{\;}^{*}\;(\text{fractional contribution of oxidase }\\ {\text{water * oxidase water}}^{18/16}\alpha ) \end{array}$$

### 2.7. Model outputs

Our model had eight oxygen outputs (see table 2), each of which was defined by moles of oxygen over the time period of measurement, and fractionation that could have occurred during loss (^18/16^*α*, ^17/16^*α* or *θ*). The first output, bound oxygen in faecal waste, was assumed to be zero moles because most dry faeces is undigested food which therefore does not interact with body water [3,22]. The second output, faecal H_2_O loss, was calculated by first determining the dry faecal output, which was calculated using the kilogram of food consumed and multiplying this by the percentage undigested (1 – *D*). Using this value, faecal water loss was calculated as [3]:


(2.12) $$\text{Dry faecal output}(kg)/(1–\text{faecal water content}){\;}^{*}\;\text{faecal water content}{\;}^{*}\;55.56$$

In this equation, faecal water content is the fraction of faecal mass that is water; we used an estimated value of 0.55 [48]. The value of 55.56 again refers to the conversion of kg to moles. No fractionation factor was applied.

The third output was transcutaneous water vapour loss. We first estimated the vapour pressure deficit (VPD) in kilopascals, representing the potential for surrounding air to hold additional moisture, using the following equation [3]:


(2.13) $$((610.78{\;}^{*}\;e^{\left((\text{Ambient Temperature [C] / (Ambient Temp [C]}+237.3){\;}^{*}\;17.2694))/1000){\;}^{*}\;(1–(rh))\right)}$$

We converted the VPD from kilopascals to mm Hg and then estimated transcutaneous water vapour loss using the following equation:


(2.14) $$\text{Days}\;*\;m^{2/3\;}*\;((k{\;}^{*}\;\text{VPD}{\;}^{*}\;\text{skin}\;H_{2}O\;\text{evaporation rate}/1000){\;}^{*}\;24/18)$$

In this equation, ‘days’ is the number of days in the time period of measurement, *m* is the body mass of the mouse and *k* is the ‘Meeh factor’, a proportionality constant to convert body mass to surface area [67]. We assumed *k* to be 1100 cm^2^/kg^2/3^, which is the midpoint of common values between 1000 and 1200 [3,68]. The ‘skin H_2_O evaporation rate’ is a taxa-specific constant; we used the value for *Peromyscus leucopus* of 0.025 mg/cm^2^/h/mm Hg [51]. The value ‘1000’ converts mg to g; ‘24’ converts hours to days; and ‘18’ converts g to moles. Transcutaneous water vapour loss was a fractionating output, and we used an ^18/16^*α* value of 0.982 [3] and a ^17/16^*α* value was calculated using the *θ* value of 0.5235 [22] and equation (2.4).

The fourth and fifth outputs were water vapour lost orally and nasally. To estimate these outputs, the moles of water vapour exhaled during breathing was calculated using the amount of air fluxed through the lungs determined using equation (2.5) and incorporating this value as follows [3]:


(2.15) $$\text{Air fluxed through the lungs }\;*\;10^{\left(0.686+0.027*(BodyTemp[C])\right)}/\;760\;/\;22.4$$

The exhaled water vapour was then divided into loss via the oral cavity (0.50) and the nasal cavity (0.50) [3]. Of the 0.50 routed to the nasal cavity, we assumed that half was retained by condensation on epithelial tissue surfaces before exhalation based on previous studies of mammal nasal heat exchange [69]. Therefore, water vapour lost nasally was 0.25 of the total exhaled water vapour. Water vapour lost nasally and orally are both fractionating outputs with a *θ* value of 0.529 [64]. However, the calculation of their ^18/16^*α* values differed. The ^18/16^*α* value for water vapour lost orally was determined via the following [62,63]:


(2.16) $$1/e^{\left((11.36{\;}^{*}\;10^{5}/BodyTemp[K{]}^{2}–4.2{\;}^{*}\;10^{2}/BodyTemp[K]–2.07)/1000\right)}$$

By contrast, the ^18/16^*α* value for water vapour lost nasally was calculated as follows [62,63]:


(2.17) $$1/e^{\left((11.36{\;}^{*}\;10^{5}/((BodyTemp[K]+AmbientTemp[K])/2{)}^{2}–4.2{\;}^{*}\;10^{2}/((BodyTemp[K]+AmbientTemp[K])/2)–2.07)/1000\right)}$$

After calculating both oral and nasal ^18/16^*α* values, their respective ^17/16^*α* values were calculated using the *θ* value (0.529) and equation (2.4).

The sixth output was exhaled CO_2_. We measured this output in ml, converted to l, divided by 22.4 to convert to moles of O_2_, then doubled the amount to calculate moles of oxygen. Exhalation of CO_2_ is a fractionating output, and we calculated an ^18/16^*α* value using the following [70]:


(2.18) $$e^{(1.66{\;}^{*}\;10^{4}/BodyTemp[K]–15.69)/1000}$$

We used this ^18/16^*α* value and a *θ* value of 0.5248 [71] to calculate a ^17/16^*α* value using equation (2.4).

The seventh output was oxygen bound in excreted urea. This was estimated (in moles) using the following equation [3]:


(2.19) $$12{\;}^{*}\;\text{Food consumed}{\;}^{*}\;f_{Protein}\;{\;}^{*}\;D{\;}^{*}\;E$$

The ‘12’ in this equation refers to the moles of O_2_ per kg of protein and is assumed not to be a fractionating output.

The eighth and final output was urinary water loss. We assumed that mice were at steady state regarding oxygen fluxes, and urinary water loss was estimated by subtracting the seven other outputs from the total inputs: urinary water loss was the value required to make the total moles of oxygen inputs and outputs equal. Urinary water loss is assumed not to be a fractionating output.

The fractional loss via each output was calculated by dividing each individual output by the summed total output. Absolute ratios of ^18^O/^16^O and ^17^O/^16^O were calculated by multiplying the fractional contribution and the respective ^18/16^*α* and ^17/16^*α* values. The absolute ratios of the outputs were calculated together because they were all derived from body water, as opposed to the inputs which were derived from either atmospheric oxygen, pre-formed water or bound oxygen. The different absolute ratios of the inputs (e.g. atmospheric oxygen) for ^18^O and ^17^O were then summed and divided by the outputs. The absolute ratios of ^18^O/^16^O and ^17^O/^16^O were then used to calculate *δ*^18^O_BW_ and *δ*^17^O_BW_ (using VSMOW), and then used in combination to calculate *Δ*′^17^O_BW_.

### 2.8. Model testing

We created a model to predict the oxygen isotope values for each blood sample. Preliminary testing indicated little effect of varying the time window (48, 96, 144, 192 h) of oxygen fluxes. Therefore, we selected 96 h for the 0.49% NaCl diet, roughly matching the expected time for complete turnover of body water in *Peromyscus* species [72,73], and 48 h for the 4% NaCl diet to account for more rapid turnover caused by the increase in water intake. In each model, input parameters were adjusted to reflect manipulations throughout the experiment (table 3).

**Table 3.** Model parameters and values for each combination of housing temperature and diet for captive deer mice.

diet	0.49% NaCl			4% NaCl		
housing temperature	25°C	18°C	15°C	25°C	18°C	15°C
relative humidity (%)	45	60	68	45	60	68
period of oxygen flux measurement prior to sampling (hours)	96	96	96	48	48	48
food carbohydrate (% mass)	67.2	67.2	67.2	65.8	65.8	65.8
food protein (% by weight)	7.4	7.4	7.4	7.7	7.7	7.7
food fat (% by weight)	25.4	25.4	25.4	26.5	26.5	26.5
food *δ*^18^O (‰)	−18.16	−16.41	−16.35	−16.41	−16.41^a^	−16.35

^a^ Food *δ*^18^O is −16.41‰ if sample was collected 30 June or −16.35‰ if sample was collected 1 July.

### 2.9. Model sensitivity and data analysis

We altered the values of model variables to the reasonably expected maximum and minimum values of our study system to assess the corresponding change in *δ*^18^O_BW_ and *Δ*′^17^O_BW_ [22]. We built a base model for a mouse housed at 25°C and consuming the 0.49% NaCl diet, using mean values for the metabolic phenotyping data (e.g. food intake, water intake and O_2_ intake; table 1). Although we used the metabolic phenotyping data to build this base model, we did not include measured values like water intake or O_2_ intake in our sensitivity analysis because the effect of these variables is evaluated via our measured isotope data. Instead, we focused on variables that we did not directly measure (e.g. *z*-value and OUF value) and fractionation factors that are not well-defined (e.g. *α* and *θ* values) to assess their effect on *δ*^18^O_BW_ and *Δ*′^17^O_BW_. We ranked each parameter and model component by the size of the effect on *δ*^18^O_BW_ and *Δ*′^17^O_BW_ values.

We conducted paired-samples *t*-tests in R [47] to compare the effect of treatments on water intake, O_2_ intake and measured *Δ*′^17^O_BW_.

## 3. Results

### 3.1. Effect of cool temperature and high salt diet

Logistical constraints prevented some sample collections and analyses: oxygen isotopes were measured for blood samples from all six sampling points (*n* = 3 mice) or from 5 points (*n* = 1), four points (*n* = 2) or three points (*n* = 2; electronic supplementary material, table S3). Of the total of 37 samples from all mice, 28 had enough volume for 2−5 distilled replicates and 9 had only enough volume for a single distillation (electronic supplementary material, table S4). Mice were sampled multiple times after being housed at 25°C and consuming the 0.49% NaCl diet: once as part of the experimental manipulations, once at the end of the acclimation period (day 1), and once at the end of the ‘reset’ period (day 42). The data collected on day 1 and day 42 were similar to the data collected during the 25°C and 0.49% NaCl manipulation: O_2_ intake = 0.97 ml * min^−1^ (±0.17), water consumption = 2.61 ml day^−1^ (±0.43), *δ*^18^O_BW_ = −1.99‰ (±0.33‰), and *Δ*′^17^O_BW_ = −90 per meg (±8 per meg). To avoid pseudoreplication, data from day 1 and day 42 were not further considered or analysed, resulting in 28 datapoints across eight mice that were analysed below.

The effects of changes in housing temperature and dietary salt content are summarized in table 4; data from 15°C and 18°C were pooled because these treatments yielded similar data (see electronic supplementary material, table S5 for more details). Mice consuming the 4% NaCl diet exhibited greater water intake than mice consuming the 0.49% NaCl diet at both 25°C (*t* = 9.72; df = 5; *p* < 0.001) and at 15°C or 18°C (*t* = 14.70; df = 5; *p* < 0.001). Across all housing temperatures, consuming the 4% NaCl diet led to water intake increases of 114−131%; by contrast, food intake, body mass and O_2_ consumption changed by <20%. Being housed at 15°C or 18°C led to a higher O_2_ intake when consuming the 4% NaCl diet (*t* = 6.91; df = 7; *p* =<0.001) compared with when mice consuming the 4% NaCl diet were housed at 25°C. Across both diets, when mice were held at 15°C or 18°C instead of 25°C, their average O_2_ intake increased by 25−52%. Cooler temperatures also increased water intake (3–25 %) and increased food intake (9–45 %).

**Table 4.** Average metabolic data (± 1 *σ*) of captive deer mice across combinations of housing temperature and diet.

diet	0.49% NaCl		4% NaCl	
housing temperature	25°C (*n* = 6)	15/18°C (*n* = 6)	25°C (*n* = 8)	15/18°C (*n* = 8)
mass (g)	19.19 ± 1.17	19.96 ± 1.01	19.12 ± 0.98	19.62 ± 1.28
O_2_ intake (ml/min)	0.95 ± 0.23	1.33 ± 0.19	0.96 ± 0.12	1.39 ± 0.18
$RQ\;(V_{CO_{2}}/V_{O_{2}})$	0.87 ± 0.01	0.88 ± 0.01	0.80 ± 0.03	0.87 ± 0.03
food intake (g)	3.48 ± 1.49	4.00 ± 0.70	3.14 ± 1.35	4.48 ± 0.98
water intake (ml)	2.42 ± 0.40	2.86 ± 0.36	5.60 ± 1.29	6.19 ± 0.74

**Figure 2.**
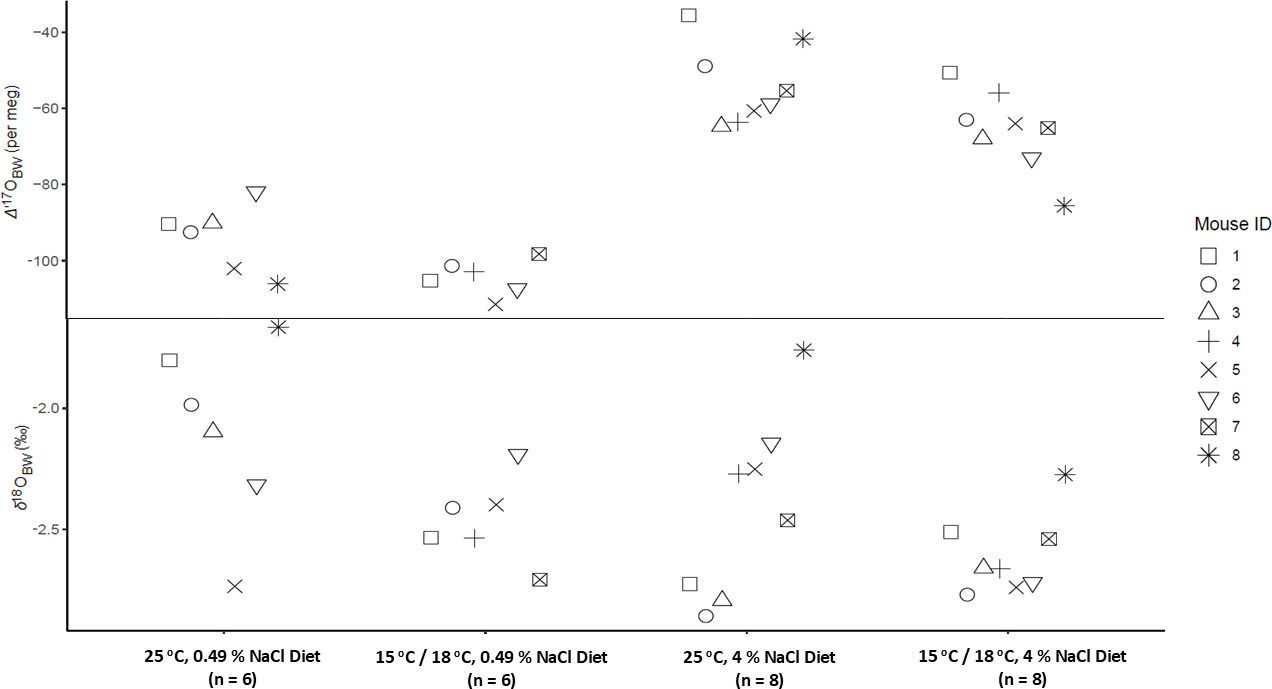
Deer mice *Δ*′^17^O_BW_ and *δ*^18^O_BW_ at different housing temperatures and consuming different diets.

Isotope analysis occurred over the span of 16 analysis runs from July to November 2023 (correction factors are in electronic supplementary material, table S6). Mice consuming the 4% NaCl diet had higher *Δ*′^17^O_BW_ than when consuming the 0.49% NaCl diet when housed at both 25°C (*t* = 6.40; df = 5; *p* < 0.001) and 15°C or 18°C (*t* = 9.93; df = 5; *p* < 0.001). Across all housing temperatures, consuming the 4% NaCl diet led to *δ*^18^O_BW_ values changing by −0.93‰ to 0.49‰, while *Δ*′^17^O_BW_ increased between 25 and 64 per meg (table 5; figure 2). Mice had a lower *Δ*′^17^O_BW_ when housed at 15°C or 18°C when consuming the 4% NaCl diet (*t* = −2.22; df = 7; *p* = 0.031) compared with when mice consuming the 4% NaCl diet were housed at 25°C. Across both diets, decreasing the housing temperature from 25°C to 15°C or 18°C resulted in a decrease of *Δ*′^17^O_BW_ of 3−44 per meg (excluding one measurement that increased by 8 per meg), while changes in *δ*^18^O_BW_ values varied from −0.73‰ to 0.34‰.

**Table 5.** Average triple oxygen isotope data (± 1 *σ*) across combinations of housing temperature and diet.

diet	0.49% NaCl		4% NaCl	
housing temperature	25°C (*n* = 6)	15/18°C (*n* = 6)	25°C (*n* = 8)	15/18°C (*n* = 8)
*δ*^17^O_BW_ (‰)	−1.21 ± 0.20	−1.40 ± 0.09	−1.33 ± 0.20	−1.45 ± 0.08
*δ*^18^O_BW_ (‰)	−2.10 ± 0.39	−2.46 ± 0.17	−2.41 ± 0.38	−2.61 ± 0.16
*Δ*′^17^O_BW_ (per meg)	−94 ± 9	−104 ± 5	−54 ± 11	−66 ± 11

### 3.2. Triple oxygen isotope modelling

**Figure 3.**
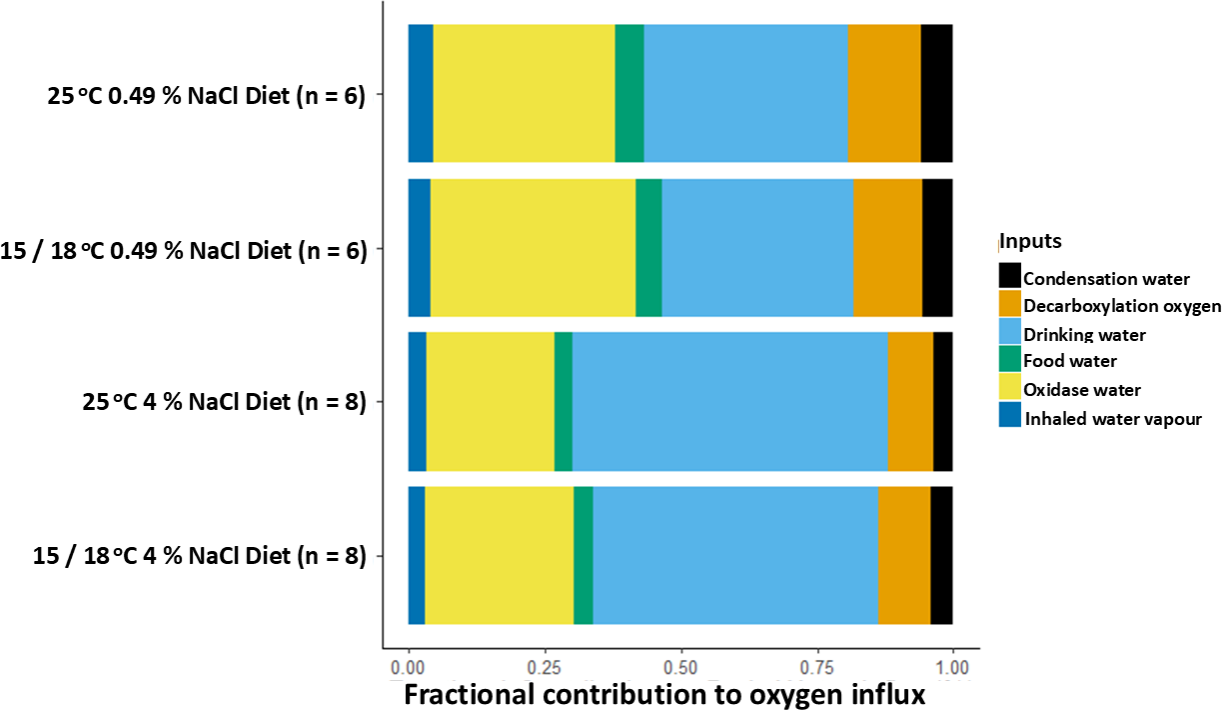
Mean fractional contributions of different oxygen influxes for deer mice at different housing temperatures and consuming different diets. Standard deviations for each influx ranged from <0.01 to 0.07 (see electronic supplementary material, table S7 for full details).

**Figure 4.**
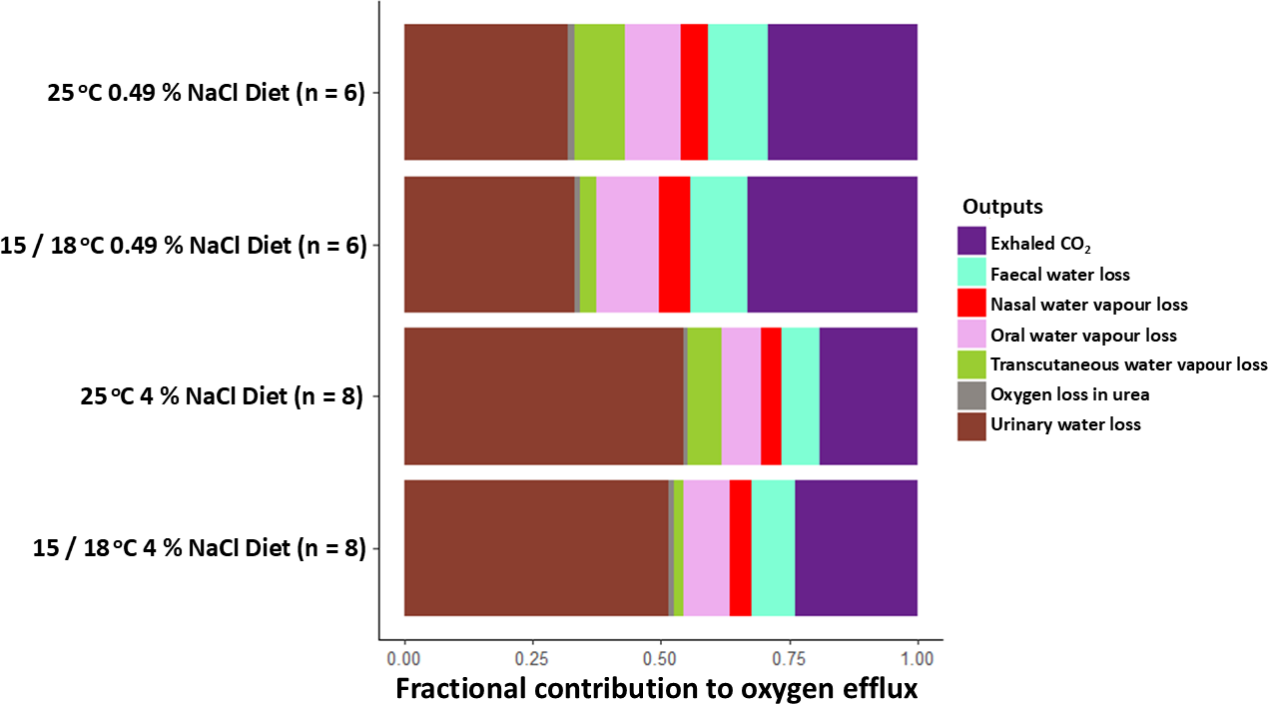
Mean fractional contributions of different oxygen effluxes for deer mice at different housing temperatures and consuming different diets. Standard deviations for each efflux ranged from <0.01 to 0.06 (see electronic supplementary material, table S7 for full details).

For mice across all temperature and diet manipulations, the two largest estimated oxygen fractional inputs were drinking water (0.35−0.58) and oxidase water (0.24−0.39; figure 3; electronic supplementary material, table S7). For all housing temperatures, model estimates indicated that when mice consumed the 4% NaCl diet, the average fractional contribution from drinking water increased by 0.17−0.20 over the value when mice consumed the 0.49% NaCl diet. For both diets, models estimated that lowering the housing temperature from 25°C to 18°C or 15°C increased the average fractional contribution of oxidase water by 0.03−0.05. For all treatments, the largest estimated oxygen fractional outputs were exhaled CO_2_ (0.19−0.34) and urinary water loss (0.31−0.54; figure 4). Across all housing temperatures, model estimates indicated that when mice consumed the 4% NaCl diet instead of the 0.49% NaCl diet, their average fractional output of urinary water loss increased by 0.18−0.23.

**Figure 5.**
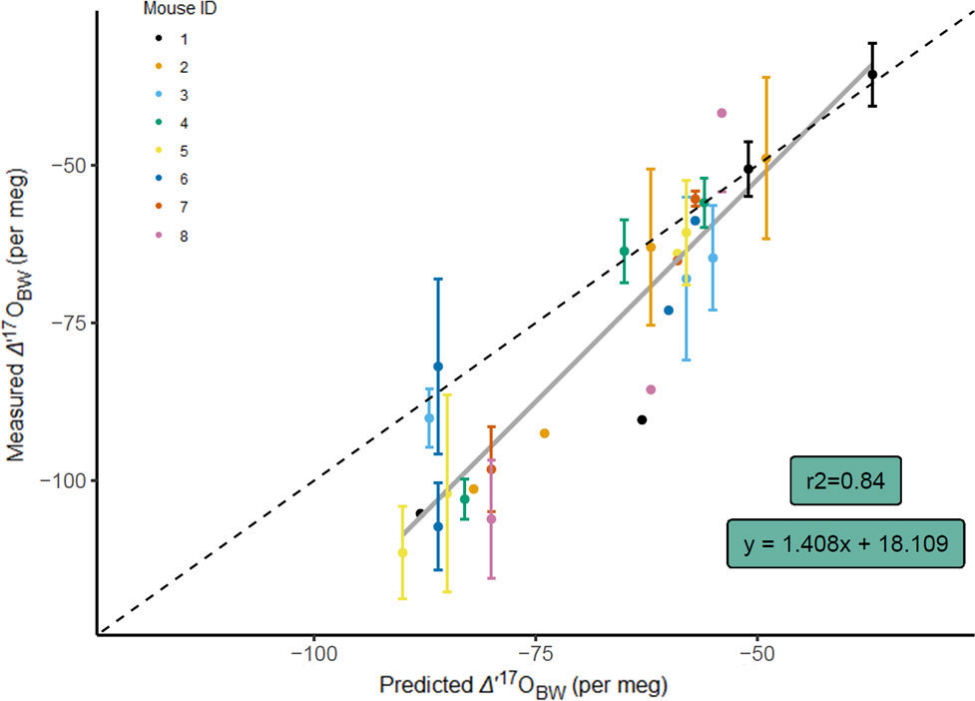
Predicted versus measured *Δ*′^17^O_BW_ for deer mice (grey line is linear regression), with 1:1 relationship (dashed line). Measurements and predictions are displayed for a total of 28 samples of eight mice. Vertical error bars (± 1 *σ*) are included for measured *Δ*′^17^O_BW_ data with ≥2 compiled measurements.

**Figure 6.**
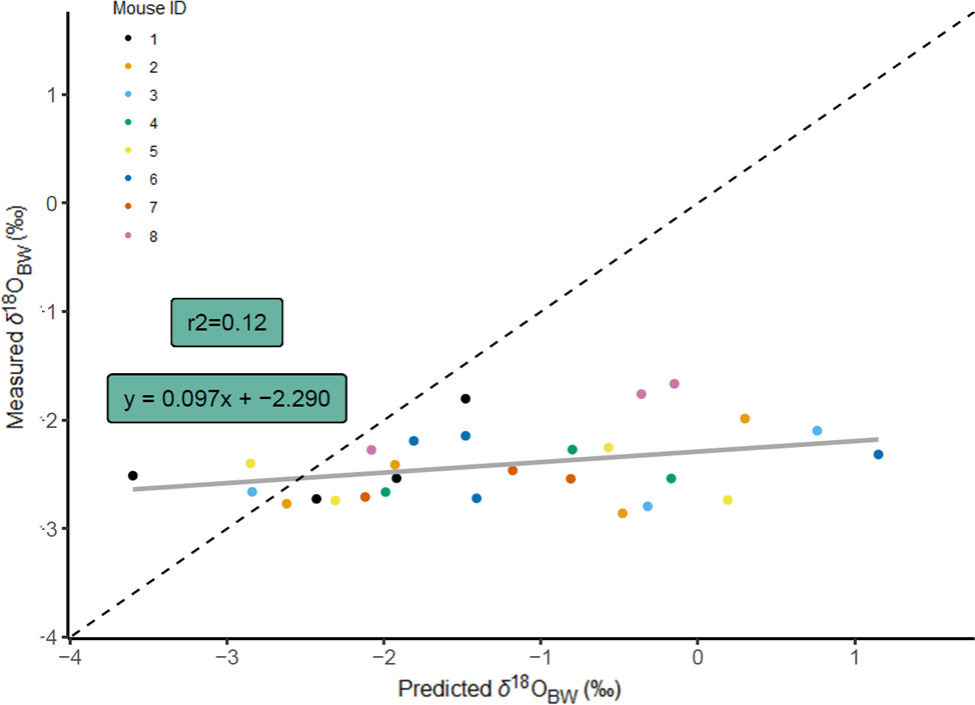
Predicted versus measured *δ*^18^O_BW_ for deer mice (grey line is linear regression), with 1:1 relationship (dashed line). Measurements and predictions are displayed for a total of 28 samples of eight mice. Vertical error bars are not included because all measured *δ*^18^O_BW_ data with ≥2 compiled measurements had standard deviations <0.3‰.

Model predictions performed well across the full range of measured values of *Δ*′^17^O_BW_ (figure 5), but performance decreased when predicting *δ*^18^O_BW_ (figure 6). Overall, the mean absolute difference between model predictions and measured values for *δ*^18^O_BW_ was 1.27‰ (± 0.97‰) and for *Δ*′^17^O_BW_ was 11 per meg (± 9 per meg). Model estimates of *Δ*′^17^O_BW_ were more accurate for mice when drinking water contributed most of the oxygen influx (i.e. mice consuming the 4% NaCl diet), with 4−7 per meg mean absolute differences. When drinking water was less important (i.e. mice consuming the 0.49% NaCl diet), mean absolute differences were 16−19 per meg (table 6).

**Table 6.** Comparison of average measured and predicted triple oxygen isotope data (± 1 *σ*) across combinations of housing temperature and diet.

diet	0.49% NaCl		4% NaCl	
housing temperature	25°C (*n* = 6)	15/18°C (*n* = 6)	25°C (*n* = 8)	15/18°C (*n* = 8)
measured *δ*^17^O_BW_ (‰)	−1.21 ± 0.20	−1.40 ± 0.09	−1.33 ± 0.20	−1.45 ± 0.08
predicted *δ*^17^O_BW_ (‰)	−0.01 ± 0.47	−1.03 ± 0.47	−0.56 ± 0.38	−1.23 ± 0.45
mean absolute difference (‰)	1.19	0.44	0.77	0.38
measured *δ*^18^O_BW_ (‰)	−2.10 ± 0.39	−2.46 ± 0.17	−2.41 ± 0.38	−2.61 ± 0.16
predicted *δ*^18^O_BW_ (‰)	0.13 ± 0.91	−1.80 ± 0.88	−0.95 ± 0.72	−2.21 ± 0.86
mean absolute difference (‰)	2.23	0.81	1.46	0.72
measured *Δ*′^17^O_BW_ (per meg)	−94 ± 9	−104 ± 5	−54 ± 11	−66 ± 11
predicted *Δ*′^17^O_BW_ (per meg)	−79 ± 9	−85 ± 4	−54 ± 8	−58 ± 4
mean absolute difference (per meg)	16	19	4	7

### 3.3. Sensitivity analysis

Skin H_2_O evaporation rate and the *z*-value had the largest effect on *δ*^18^O_BW_, while *z*-value and OUF had the largest effect on *Δ*′^17^O_BW_ (table 7). Additionally, the *δ*^18^O of atmospheric O_2_ (*δ*^18^O_BW_ rank: 6; *Δ*′^17^O_BW_ rank: 10) and the ^18/16^*α* value used in relation to oxidase water (*δ*^18^O_BW_ rank: 5; *Δ*′^17^O_BW_ rank: 6) had large effects on both *δ*^18^O_BW_ and *Δ*′^17^O_BW_.

**Table 7.** Deer mouse model sensitivity analysis of variation to individual parameter values and certain model components.

		sensitivity test value range		change in *δ*^18^O_BW_ (‰) or *Δ*′^17^O_BW_ (per meg) values		rank of effect on *δ*^18^O_BW_ or *Δ*′^17^O_BW_ values	
parameter	base model value	low	high	*δ*^18^O_BW_ change	*Δ*′^17^O_BW_ change	*δ*^18^O_BW_ rank	*Δ*′^17^O_BW_ rank
*model parameters*							
faecal H_2_O content (%)	55	30	90	0.00	0	21	26
oxygen utilization fraction/fraction of O_2_ used (%)	25	5	45	1.70	38	8	2
*z*-value (‰)	10.5	4	12	3.01	40	2	1
body temperature (C)	38	34	40	0.50	1	14	24
Meeh factor (*k*)	1100	900	1300	0.31	0	16	26
skin H_2_O evaporation rate (mg/cm^2^/h/mm Hg)	0.025	0.001	0.1	5.12	4	1	13
relative humidity (%)	45	0.0	100	2.29	2	4	17
environmental temperature (C)	25	0	40	1.35	2	10	16
pre-formed water *δ*^18^O (‰)	−4	0	−6	1.63	7	9	11
pre-formed water *Δ*′^17^O (per meg)	0.02	0	40	0	12	21	8
atmospheric O_2_ *δ*^18^O (‰)	24.046	20	27	1.87	9	6	10
atmospheric O_2_ *Δ*′^17^O (per meg)	−0.441	−0.52	−0.36	0	29	21	4
relative digestibility (%)	85	40	90	2.88	2	3	17
water nozzle leakage (% of water intake estimated to be leakage)	10	0	30	0.39	11	15	9
energy extraction efficiency (%)	100	80	100	1.02	1	11	25
food associated free H_2_O content (%)	9	0	25	1.72	5	7	12
food *δ*^18^O (‰)	−16.41	−25	0	0.82	4	12	14
food *Δ*′^17^O (per meg)	30	0	50	0	1	21	23
*δ*^18^O of the cellulose of corn ingredients in diet	26	22	30	0.27	2	18	20
*δ*^18^O of the cellulose of wheat ingredients in diet	26.9	22	30	0.31	2	16	17
*δ*^18^O of the cellulose of soybean ingredients in diet	25.9	22	30	0.22	2	19	22
*model components*							
*θ*_Exhaled CO2_ _– body water_^a^	0.5248	0.522	0.526	0	32	21	3
*θ*_Cellulose – pre-formed water_ ^b^	0.525	0.525	0.53	0	28	21	5
*θ*_Transcutaneous water vapour loss – body water_ ^c^	0.5235	0.516	0.525	0	13	21	7
^18/16^ *α*_Transcutaneous water vapour loss – body water_ ^d^	0.982	0.976	0.99	0.74	2	13	21
*θ*_Lung absorbed O2 – atmospheric O2_ ^e^	0.5179	0.5166	0.519	0	3	21	15
^18/16^*α*_Lung absorbed O2_ _– atmospheric O2_ ^f^	0.994	0.99	1	2.07	27	5	6
fractional split of moles of exhaled water vapour from breathing assigned to oral vapour water loss and nasal water vapour loss	1/2 and 1/4	1/16 & 11/16	7/16 and 5/16	0.02	0	20	26

^a^ Sensitivity analysis low determined from Hofmann *et al*. [74].

^b^ Sensitivity analysis range determined from Pack *et al*. [30].

^c^ Sensitivity analysis low value determined from Feng *et al*. [27] and Landais *et al*. [75].

^d^ Sensitivity analysis low value determined from Bryant & Froelich [16].

^e^ Sensitivity analysis range determined from Luz & Barkan [61].

^f^ Sensitivity analysis range from Hu *et al*. [22].

## 4. Discussion

Our findings are encouraging to the continued development of triple oxygen isotope analysis to study animal biology, specifically metabolism and water balance. In support of our predictions, mice increased their drinking water intake when consuming a high-salt diet, driving their *Δ*′^17^O_BW_ towards the *Δ*′^17^O value of the drinking water source. At cooler housing temperatures, mice increased their oxygen consumption to meet the energetic demand of thermoregulation, and we infer that this resulted in greater production of oxidase water, driving their *Δ*′^17^O_BW_ value towards the *Δ*′^17^O value of atmospheric oxygen. We also found that our modelled *Δ*′^17^O_BW_ predictions were accurate: all predicted values were within 30 per meg of measurements, and 43% (12/28) were within 5 per meg of measured values, which is similar to expected measurement uncertainty.

Our results show that a triple oxygen isotope approach has the unique ability to infer variation in water influxes. The disparity between the *Δ*′^17^O values of oxidase water and other inputs such as drinking water is key to modelling *Δ*′^17^O_BW_ [7]. Importantly, even wide-ranging species likely experience only small variation in the *Δ*′^17^O value of their drinking water. For example, tap water *Δ*′^17^O values throughout the United States range from −6 to 43 per meg (−0.006−0.043‰ [76]). An exception is animals reliant upon highly evaporated surface waters, such as African ungulates, as these sources can have altered *Δ*′^17^O [27]. Similarly, the incorporation of atmospheric oxygen into oxidase water is common across animals, meaning that the related *Δ*′^17^O values (approx. −441 per meg) should reflect metabolism across taxa [7].

Although the changes in water intake and metabolic rate observed in our study were induced by experimental manipulations, these shifts are comparable with those experienced by free-ranging animals because of seasonal or life-history variation. For example, the wild ash-grey mouse (*Pseudomys albocinereus*) of Australia—with a similar body mass as our study species—doubles its water intake from the dry to the rainy season [77]. This large increase in water intake is similar to the effect of consuming the 4% NaCl diet in our study, which resulted in a 25−64 per meg increase in *Δ*′^17^O_BW_. In comparison, the cooler housing temperatures in our study increased mouse metabolic rate by 25−52%, variation that free-ranging animals may even exceed. For example, female long-tailed pocket mice (*Chaetodipus formosus*) of the Mojave Desert (NV, USA)—also with a body mass similar to our study species—double their metabolic rate when transitioning from pregnancy to lactation [78]. Although the elicited change in metabolic rate was smaller in our study, it still yielded declines in *Δ*′^17^O_BW_ as large as 44 per meg.

We found that the biggest oxygen influxes were from drinking water and oxidase water, and that the biggest effluxes were from urinary water loss and exhaled CO_2_. Although some of our fluxes are estimated, similar fractional contributions to water influx and efflux were demonstrated for drinking water, oxidase water and urinary water loss in studies of other captive *Peromyscus* species [79,80]. We can also compare our results with a study which measured *Δ*′^17^O_BW_ in bioapatite from tooth enamel of wild, similar-sized rodents (wood mouse and striped field mouse, *Apodemus sylvaticus* and *Apodemus agrarius* [27]). That study estimated greater influxes of oxidase water (0.48–0.66) and effluxes of exhaled CO_2_ (0.36–0.58) than we found, potentially illustrating the effects of higher metabolic rates at colder temperatures (estimated at 8°C) and reduced intake of drinking water as compared with captive animals with ad libitum access. The fractional contributions of oxidase water input and exhaled CO_2_ output are particularly important to interpreting measurements of *Δ*′^17^O_BW_ because both are fractionating fluxes. Notably, the model developed by Whiteman *et al*. [7] to interpret measurements of *Δ*′^17^O_BW_ did not include any oxygen outputs, like exhaled CO_2_, or incorporate *θ* values, and this may have contributed to a consistent and substantial bias in model predictions of ≤ approximately 65 per meg. This is supported by our sensitivity analysis that demonstrated that oxygen outputs and *θ* values, such as the *θ* value related to exhaled CO_2_, have critical effects on model predictions and that not incorporating these components can contribute to significant model error. Our results demonstrate that accurate and realistic estimates of fractional contributions of all fluxes, especially fractionating fluxes (e.g. transcutaneous water loss, inhaled water vapour, nasal water vapour loss), are critical to interpretating *Δ*′^17^O_BW_.

Despite the ability of the model to predict *Δ*′^17^O_BW_, the model was less accurate when predicting *δ*^18^O_BW_. Like the findings of our study, previous assessments of the *δ*^18^O_BW_ estimates from the Kohn [3] model demonstrated predictions commonly differing from measured values by >1‰ [81,82], which were partly attributed to difficulties in predicting fractionating effluxes (e.g. transcutaneous water vapour loss) and in assigning values to parameters related to these effluxes [81,82]. This is consistent with our sensitivity analysis indicating the importance of less constrained *α* values such as those associated with transcutaneous water vapour loss. As previous studies noted [7,22,30], the advantages of using *Δ*′^17^O for modelling oxygen fluxes are that mass-independent fractionating processes are generally easier to constrain than mass-dependent processes, and that the latter infrequently exhibit slopes differing from 0.528. The greater ease in predicting *Δ*′^17^O_BW_ compared with *δ*^18^O_BW_ is apparent in our sensitivity analysis showing that many of the parameters that hinder our ability to accurately predict *δ*^18^O [81] are largely reduced for *Δ*′^17^O. For example, skin H_2_O evaporation rate is one of the most sensitive parameters for predicting *δ*^18^O_BW_ but does not appear to be important for estimating *Δ*′^17^O_BW_ (table 7).

Modelling of *Δ*′^17^O_BW_ is still in its infancy and therefore it is important to integrate our study with previous *Δ*′^17^O_BW_ modelling. Several previous studies have focused on measurements of fossilized biological samples, with the premise that the inferred *Δ*′^17^O_BW_ value was integrated over the lifespan of the animal, enabling the back calculation of *Δ*′^17^O of atmospheric oxygen and paleoclimate applications (e.g. [22,30]). In comparison, we used distilled blood samples and assumed that the *Δ*′^17^O_BW_ values were representative of the period of body water turnover. This focus on shorter time periods in living animals has potential applications in wildlife research and conservation because small samples (e.g. ≤50 µl of blood) from single capture events are more feasible to collect than the repeated sampling required for the DLW method and can provide insight regarding metabolism and reliance on drinking and food water for specific time periods of an animal’s life.

Our models also differ from other flux-based models [3,22], which assumed that all bound oxygen in food was used for synthesizing condensation water. By contrast, we assume that bound oxygen in protein and lipids initially appear in CO_2_, not condensation water. This assumption is because, unlike carbohydrates, protein and lipids enter the pathway of aerobic oxidation at steps downstream of the reaction that synthesizes condensation water using bound oxygen from food (see electronic supplementary material, S1). For example, fatty acids enter the pathway via conversion to acetyl-CoA (i.e. beta-oxidation [2]), which is downstream of the synthesis of phosphoenolpyruvate in glycolysis, a reaction that yields condensation water [2]. We also assume that for glucose, two of the bound oxygen in this molecule appear in condensation water and four appear in CO_2_, based on the biochemical structures of intermediates during aerobic oxidation [6] (also see electronic supplementary material, S1). Our model also differs from previous efforts in several estimates of water losses because we did not use a generalized value of 1 mg cm^−2^ h^−1^ for calculating transcutaneous water vapour loss [3,22,27]. While this variable has not been measured for many species, there is consistent variation in the skin H_2_O evaporation rate among broad groupings of taxa, such that specific values can be estimated for a given study species [51]. Previous *Δ*′^17^O_BW_ studies have also assumed that urinary water loss was 25% of the total water inputs, and that differences in oxygen inputs and outputs were due to water loss for thermoregulation via sweating and panting. However, taxa-specific information can constrain these assumptions. In our experiment, we expected water loss for thermoregulation via sweating and panting to be zero because temperature never increased above the predicted upper critical temperature for deer mice (estimated between 35°C and 37°C [34]) and because, in general, mice do not sweat [83,84]. This allowed us to eliminate a final adjustment to inputs and outputs that was required in previous models, but did not have a biological basis. Instead, we used the difference between input and output to estimate urinary water loss, allowing this variable to change as animals drank more water. Feng *et al*. [27] used a similar approach to our study for estimating urinary water by determining a residual from the inputs and outputs (assuming steady state) but combined urinary water loss and faecal water loss.

The *θ* value between cellulose and pre-formed water can have an important influence on the effect of condensation water (via bound oxygen) on *Δ*′^17^O_BW_, and was estimated as 0.525−0.530 [30], with subsequent models using the midpoint (0.5275) of this range [22,27]. We used 0.525, the lower point of this range, after preliminary trials suggested that this was more consistent with our data. In addition, 0.525 is closer to the fractionation value (<0.5216) between leaf water and pre-formed water [75]. Notably, previous studies have indicated that *θ* values can be underestimated due to errors related to unaccounted for pressure baseline offsets during measurements [85]. Regardless, we caution that this remains a limitation of this modelling approach until further investigation can be conducted regarding this *θ* value.

Our results identify key limitations that remain for applications of *Δ*′^17^O_BW_ modelling. Specifically, our sensitivity analysis revealed that the *z*-value, OUF value and exhaled CO_2_ *θ* value had the strongest influence on *Δ*′^17^O_BW_, but these parameters are poorly constrained. The *z*-value and OUF value are critical parameters because they are vital for estimating the *δ*^18^O_BW_ and *Δ*′^17^O_BW_ of oxidase water. Specifically, the ^18/16^*α* value for inhaled atmospheric oxygen, to form oxidase water, is calculated using the assigned *z*-value and OUF value, and our sensitivity analysis revealed that this ^18/16^*α* value had a large effect on both modelled *δ*^18^O_BW_ and *Δ*′^17^O_BW_. OUF values can vary widely in animals (10–40% [36,86,87]) and *z*-values have primarily been studied in humans [59,60]. The reduced performance of our model when the contribution from oxidase water increased (as input from drinking water decreased) may reflect the uncertainty of parameters like the *z*-value and OUF. Future captive studies are needed with a diverse range of taxa to better understand the range of OUF and *z*-values among different taxa. These studies can be challenging because they require analysis of breath samples [59,60], which can be difficult to obtain under normal, baseline breathing without conditioning the animal to this type of sampling. In addition, the contribution of exhaled CO_2_ to oxygen effluxes similarly increased when the contribution from oxidase water increased. Like *z*-values and OUF values, the *θ* value related to exhaled CO_2_ requires further exploration [71,74,88]. Potential application of different modelling approaches such as a Bayesian approach may aid in estimating these parameters, although more data on parameters like the *z*-value is needed to inform these types of models. Our sensitivity analysis revealed that the *z*-value, OUF value and exhaled CO_2_ *θ* value had the strongest influence on *Δ*′^17^O_BW_, so further investigation of these variables is critical to improving *Δ*′^17^O_BW_ modelling and understanding the cascading effects of the *z*-value and OUF value on calculated fractionation factors remains a major limitation of this modelling approach.

Future studies should continue to explore how fundamental biological processes influence *Δ*′^17^O_BW_ and assess the range and variation of *Δ*′^17^O_BW_ of free-ranging wildlife. For example, captive studies of growing, juvenile animals would allow assessment of model performance in non-steady state scenarios, and comparisons of males and females could explore effects of sex-specific physiological processes (e.g. lactation). Captive studies of taxa that differ in key oxygen fluxes are also needed: for example, compared with mammals, avian fauna typically have higher metabolic rates and herpetofauna have lower metabolic rates, and both have different water balance strategies (e.g. uric acid excretion rather than urea [4,89]). Models for carnivores should also be refined to better understand the effects of prey body water intake, and models of herbivores should be refined to assess the capabilities of *Δ*′^17^O_BW_ modelling when accounting for plant waters (e.g. leaf water), which can have wide ranging *Δ*′^17^O values [21]. In addition to captive experiments, studies can test for differences in *Δ*′^17^O_BW_ between or within populations based on broad expectations for important physiological dynamics, such as seasonal variation in metabolic rate or access to environmental water sources. Longitudinal studies of free-ranging animals would be especially informative, to assess the role of environmental seasonality in parameters such as ambient temperature and relative humidity on modelled *δ*^18^O_BW_ and *Δ*′^17^O_BW_. Such field studies can include large sample sizes to improve inferential power because a *Δ*′^17^O_BW_ approach only requires a single sample, which broadens its application to large and elusive species that are difficult to capture. Studying the water intake and metabolism of these species was previously difficult using common methods like the DLW approach because of the need for multiple capture events and injection of costly isotope tracers.

## Data Availability

Data and relevant code for this research work are included in the electronic supplementary material and stored in GitHub [90], and have been archived within the Zenodo repository [91]. Supplementary material is available online [92].
